# Light chain deposition disease without proteinuria

**DOI:** 10.1002/jha2.102

**Published:** 2020-09-16

**Authors:** Shinichi Mizuno, Chigusa Kitayama

**Affiliations:** ^1^ Department of Nephrology Japan Community Health Care Organization Sendai Hospital Sendai Japan

**FIGURE 1:**
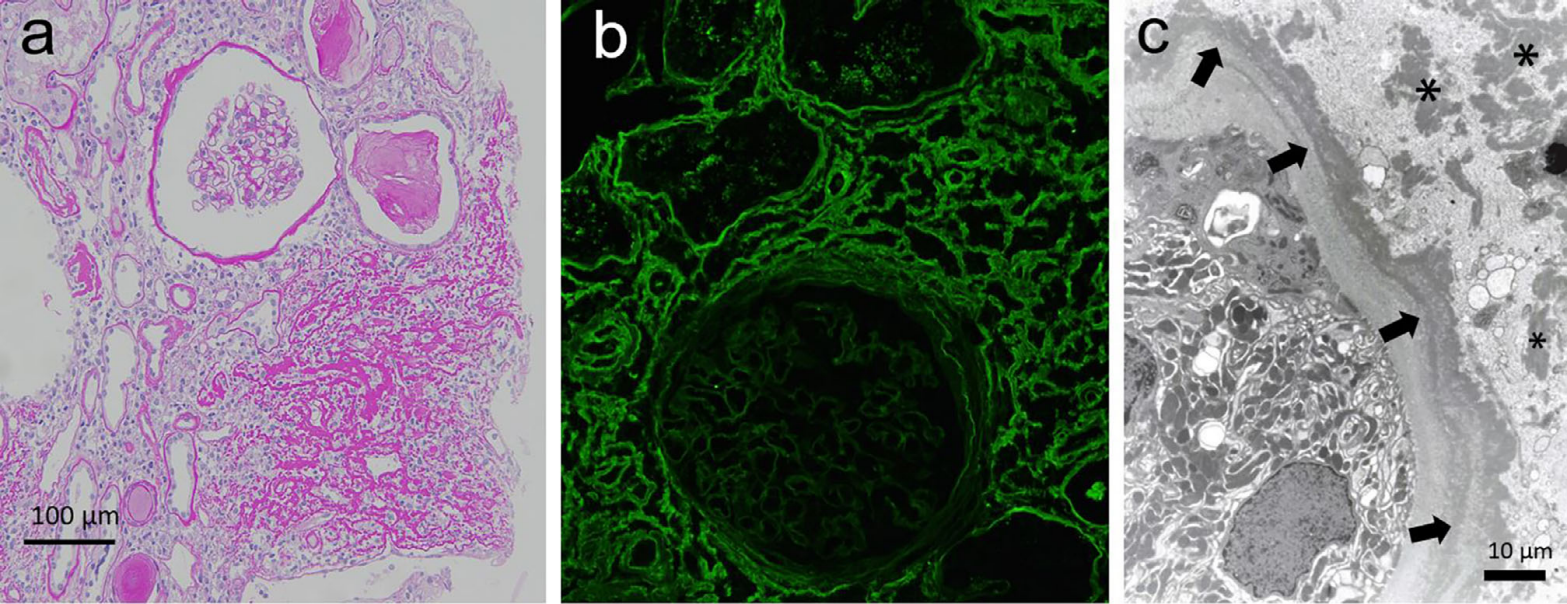
Pathological findings of renal biopsy. A, Light microscopy shows strongly eosinophilic deposits on the interstitium and tubular basement membranes (periodic acid‐Schiff staining; original magnification, 200×). B, Kappa light chain staining is positive on the interstitium and tubular basement membranes. No evidence of glomerular deposits is found (original magnification, 200×). C, The electron microscopy shows nonorganized deposits along the tubular basement membranes (arrows) and on the interstitium (asterisks) (original magnification, 3000×)

An 80‐year‐old man was admitted with a high serum creatinine level of 2.8 mg/dL. Laboratory examinations revealed a serum IgG level of 3075 mg/dL and serum immunoelectrophoresis detected IgG‐kappa monoclonal protein. The level of serum kappa light chain was 270 mg/L and that of lambda chain was 34 mg/L. Urinalysis demonstrated proteinuria of only 0.1 g/day without hematuria, but urinary β2‐microglobulin was abnormally high at 19 500 μg/L (normal range, <230). Renal biopsy revealed strongly eosinophilic deposits on the interstitium and tubular atrophy with tubular basement membrane (TBM) thickening (Figure 1A). Amyloid deposits were not observed on direct fast scarlet staining. Only kappa light chain deposits on the TBM and interstitium were observed by immunofluorescence, but there was no evidence of glomerular deposits (Figure 1B). On electron microscopy, nonorganized deposits were noted along the TBM and on the interstitium (Figure 1C). We diagnosed the patient with light chain deposition disease (LCDD) by monoclonal gammopathy of renal significance (MGRS) because the bone marrow test demonstrated only 0.4% plasma cells.

Recently, the predictors of MGRS before renal biopsy have been reported as hematuria and proteinuria. LCDD is a rare complication of paraproteinemia including MGRS, characterized by massive proteinuria and monoclonal light chain deposits along glomerular and TBMs; however, the rare variant of tubulointerstitial limited LCDD without proteinuria is not well known. MGRS lesions localized to the tubulointerstitium are likely to be missed because of lack of hematuria and proteinuria before renal biopsy. In cases of renal dysfunction with monoclonal protein absent proteinuria or hematuria, abnormal levels of urinary β2‐microglobulin might be helpful tool for suspecting MGRS.

